# Detection of coagulopathy in paediatric heart surgery [DECISION study]: study protocol

**DOI:** 10.1186/s12878-015-0030-8

**Published:** 2015-08-19

**Authors:** Wendy Underwood, Chris A. Rogers, Zoe Plummer, Barnaby C Reeves, Massimo Caputo, Peter Murphy, Karen Sheehan, Jessica Harris, Lucy Culliford, Andrew Mumford

**Affiliations:** School of Cellular and Molecular Medicine, University of Bristol, Bristol, UK; Clinical Trials and Evaluation Unit, School of Clinical Sciences, University of Bristol, Bristol, UK; The Bristol Heart Institute, University Hospitals Bristol NHS Foundation Trust, Bristol, UK; Bristol Royal Hospital for Children, University Hospitals Bristol NHS Foundation Trust, Bristol, UK

**Keywords:** Heart surgery, Paediatric, Coaugulation, Thromboelastometry, Rotational TEM, Cardiopulmonary bypass

## Abstract

**Background:**

Each year in the UK, ≈3000 children undergo major cardiac surgery requiring cardiopulmonary bypass. Approximately 40 % of these experience excessive bleeding necessitating red cell transfusion or treatment with other blood components. A further 40 % receive blood components because of the perception by clinicians that the risk of bleeding is high. Excessive bleeding and treatment with red cell transfusion or blood components are associated with post-operative complications such as infection and renal injury and are independently associated with increased morbidity and mortality.

Abnormalities in blood coagulation are a major cause of excessive bleeding after cardiac surgery in children. However, the extent of these abnormalities varies between children and their characteristics may change rapidly during surgery. In adults undergoing cardiac surgery, rapid testing of blood coagulation using techniques such as thromboelastometry may assist the selection of appropriate blood component treatments. In some sub-groups of adults, this improves clinical outcomes. Rapid testing of blood coagulation in children undergoing cardiac surgery has not been evaluated fully.

**Methods/Design:**

The DECISION study is a prospective, single-centre, observational study that aims to assess the utility of rapid testing of blood coagulation in children undergoing cardiac surgery. This will be achieved by testing blood samples from 200 children obtained immediately before, and after cardiac surgery. The blood samples will be analysed in parallel using thromboelastometry and reference laboratory tests of blood coagulation. The primary clinical outcome will be clinical concern about bleeding, defined as a composite of either excessive blood loss or the use of a pro-haemostatic treatment outside of standard treatment protocols because of perceived high risk of excessive bleeding. The reference laboratory test results will be used to describe the patterns of abnormalities in blood coagulation in children and will be compared to the thromboelastometry test results to determine the diagnostic accuracy of the thromboelastometry tests. We will estimate how well the reference and thromboelastometry test results predict clinical concern about bleeding.

**Discussion:**

The DECISION study will identify the most useful thromboeastometry tests of blood coagulation for the prediction of excessive bleeding in children after cardiac surgery and will inform the design of future randomised controlled trials.

**Trial registration:**

The trail was registered as ISRCTN55439761 on 23^rd^ April 2015.

## Background

Better blood and blood component transfusion is a longstanding NHS priority that is highly pertinent to cardiac surgery since the 40,000 cardiac surgery procedures in the UK each year account for 18 % of all NHS blood components used. This statistic includes 3,000 children who undergo cardiac surgery requiring cardiopulmonary bypass (CPB) and who have a 30-day mortality of 4 % and post-operative morbidity from organ injury or sepsis of about 25 % [1]. Excessive bleeding occurs in 40 % of children after cardiac surgery and is typically treated by transfusion of red cells and other blood components such as fresh frozen plasma, cryoprecipitate or platelet concentrates. However, in current NHS practice, a further 40 % of children receive blood components empirically at the end of surgery in an attempt to prevent excessive bleeding [2]. Decisions to administer blood components are not standardised and there is marked variation between NHS centres in the prevalence of excessive bleeding and in blood component use [2]. Excessive bleeding and transfusion of red cells or other components are independently associated with increased risk of post-operative morbidity and mortality arising from adverse events such as sepsis and kidney, brain and lung injury [1]. Adverse events have a significant impact on children and their families and substantially increases healthcare costs.

A major cause of excessive bleeding in children after cardiac surgery is abnormality in the blood clotting system. This is typically complex and multi-factorial and may be influenced by blood clotting abnormalities that pre-exist in the child. However, blood clotting is also affected by anaesthetic techniques that are essential for cardiac surgery to be performed. Therefore, blood clotting abnormalities vary markedly between children and change within individual children during the course of surgery [3–5]. Amongst the range of possible blood clotting abnormalities, low platelets and low fibrinogen have been most commonly observed in small single-centre or single-diagnosis groups of children and were associated with excessive bleeding [3–5]. Associations between these blood clotting abnormalities and excessive bleeding have not been tested in larger unselected cohorts.

Since different blood clotting abnormalities usually require different blood components, treatments to prevent or treat bleeding must be selected quickly and must be individualised to each patient. In adults undergoing cardiac surgery, this is currently assisted by rapid testing of blood clotting using techniques such as thromboelastometry (TEM), which is usually performed as a point-of-care test. TEM may yield diagnostically useful information about the characteristics and severity of blood clotting abnormalities during or after cardiac surgery. TEM test results are typically incorporated into simple treatment algorithms to assist selection of the most appropriate blood component or pharmaceutical treatments to reverse the blood clotting abnormality. Individualised treatment of blood clotting abnormalities informed by rapid test results has been shown to reduce red cell and blood component use in several clinical trials in adults [6–8] and to improve clinical outcomes in some patient groups [9].

Although TEM testing of blood clotting is an effective clinical strategy in adults undergoing cardiac surgery, the role of TEM testing in children is less clearly defined. In part this is because existing evidence suggests that the changes in blood clotting that develop in children during cardiac surgery are qualitatively different to those in adults and develop with different kinetics [3–5]. Consequently, diagnostic inferences from the results of TEM tests in adults cannot necessarily be extended to children and treatment decisions based on these results may not be valid. Although TEM tests are now performed after cardiac surgery in about 25 % of children in NHS hospitals [2], testing is not standardised and treatment decisions guided by TEM results have not been validated.

The *Detection of Coagulopathy in Paediatric Heart Surgery* (DECISION) Study has been developed to better define the clinical utility of blood clotting testing in children undergoing cardiac surgery and to identify areas for evaluation in future randomised controlled trials. It is anticipated that improvements in the diagnosis of blood clotting abnormalities in children will assist selection of appropriate blood components to prevent or treat excessive bleeding which may reduce blood component use and improve clinical outcomes after cardiac surgery.

## Methods and design

### Aims and objectives

The overall aim of the study is to evaluate whether TEM testing for blood clotting abnormalities in children before or after cardiac surgery will improve prediction of excessive bleeding after cardiac surgery.

This may confer direct clinical and economic benefits in two ways; 1) test results may improve selection of the best treatments for children with blood clotting abnormalities to prevent or stop excessive bleeding, and, b) test results may prevent the use of unnecessary treatments in children who do not have blood clotting abnormalities and are at low risk of excessive bleeding.

**Fig. 1:**
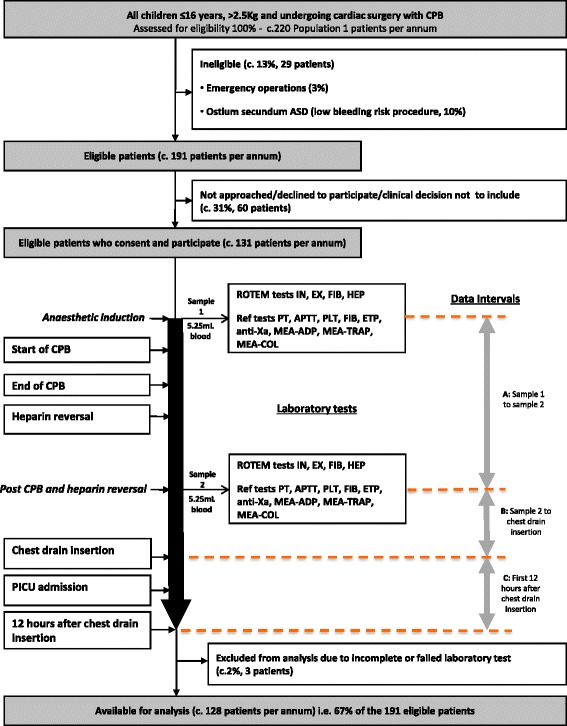
Study Schema

The study objectives are as follows:

1. To describe the prevalence of the different types of blood clotting abnormality in children before and after cardiac surgery.
2. To estimate the extent to which the TEM and reference laboratory test results predict (a) clinical concern about bleeding after cardiac surgery and (b) different types of blood clotting abnormality and clinical concern about bleeding after cardiac surgery.
3. To estimate the diagnostic accuracy of TEM test results vs. reference laboratory tests for the different types of blood clotting abnormality identified (a) before and (b) after cardiac surgery.
4. To investigate the agreement between the treatment recommended by the results of the reference tests and the treatment recommended by the TEM test results(see Figure 1 for study schema)

### Study design

Prospective, single-centre, observational cohort study (see Figure 1 for study schema).

### Study population

Children at the Bristol Royal Hospital for Children at high or moderate risk of bleeding after cardiac surgery.

### Consent

For all potential participants younger than 11 years old, the parents or guardians gave written consent after eligibility checking. For potential participants between 11 and 15 years old, signed assent was obtained from the participants and written consent from parents or guardians. The participants who were16 years old gave written consent. The study was conducted after UK Research Ethics Committee approval (NRES Committee London - City & East 13/LO/0504) in accordance with the Declaration of Helsinki.

#### Inclusion criteria

Participants may enter the study if ALL of the following apply:

1. Cardiac surgery requiring CPB (thereby at high or moderate bleeding risk)
2. Age ≤16 years old
3. Weight >2.5 Kg

#### Exclusion criteria

Participants may not enter the study if ANY of the following apply-

1. Emergency cardiac surgery
2. Unable to give informed consent (16 year olds only)
3. In foster care and parents/guardians unavailable to consent

### Study interventions

1. Two 5.25 ml blood samples will be taken from existing intravascular lines at two time-points: Sample 1, immediately following anaesthetic induction.
2. Sample 2, *after* cessation of CPB, heparin reversal and other routinely performed interventions that may influence blood clotting such as cell saver return but *before* the start of the return of pump blood (see below).

The volume of the blood required may, in exceptional circumstances, be tolerated poorly by participants with small circulating volumes or critical cardiovascular function. In order to minimise the risk of harm to participants, the clinicians with responsibility for the participants will be instructed to consider the consequence of taking each blood sample on a case-per-case basis and will not take a blood sample if this is considered to be detrimental to the participant. The clinician will be asked to sign the case report (CRF) form to indicate they agree to each blood sample being taken.

### Primary outcomes

The primary outcome is clinical concern about bleeding. This outcome will be determined for the following time intervals:

Interval A: The period between sample 1 and sample 2

Interval B: The period between sample 2 and chest drain insertion.

Interval C: The first 12 h after chest drain insertion.

Clinical concern about bleeding is defined as:

1. High blood loss. Chest drain volume of either >5 ml/Kg/hr in any 1 h interval or >3 ml/Kg/hr for 3 consecutive hours (Hazinski guidelines [7]) in interval C.
  OR
2. Any non-routine pro-haemostatic treatment given in response to bleeding by the clinical team. This is defined as any of the following: protamine given after initial heparin reversal; fresh frozen plasma (FFP); cryoprecipitate; platelet concentrates; any anti-fibrinolytic drug; activated recombinant factor VII (rFVIIa); fibrin sealant or fibrinogen concentrate.

The composite endpoint is necessary because of two important constraints:

- Blood loss is readily quantified after chest drain insertion by measuring drain volume. However, in intervals A and B (before chest drain insertion), measurement of blood loss is not feasible.
- Non-routine pro-haemostatic therapies are usually given soon after the start of abnormal bleeding and, if effective, prevent further bleeding. Measuring only ‘high blood loss’ would result in the primary endpoint of excessive bleeding not being identified in these instances.

There are two other circumstances in which clinicians administer pro-haemostatic therapies during or after cardiac surgery in children:

1. As part of our standard institutional protocols for all children in the study population. These are termed ‘routine’ pro-haemostatic therapies and include interventions such as cryoprecipitate infusion and the administration of protamine to reverse the effects of heparin at the end of surgery.
2. In response to a perceived risk of excessive bleeding but before excessive bleeding starts. These are termed ‘non-routine prophylactic’ pro-haemostatic therapies and are usually given during interval B.

Clinicians will be asked to classify all pro-haemostatic treatments in the study intervals A-C into one of three categories i) ‘routine’, ii) ‘non-routine in response to bleeding’; or ‘non-routine prophylactic’ on the study CRFs. The ‘routine’ and ‘non-routine prophylactic’ pro-haemostatic treatments will not be considered part of the primary outcome.

In some children, blood from the CPB circuit (pump blood) is re-infused during interval B and C requiring additional protamine to be administered to reverse the heparin contained in this blood. We have considered this protamine dose to be part of the ‘routine’ management. Re-infusion of pump blood will be recorded on the CRF but the additional protamine will not be classified as part of the primary clinical outcome.

### Secondary outcomes

Secondary clinical outcomes will be collected for all intervals as follows:

- Red cell transfusion expressed as any vs. none and total red cell transfusion volume.
- FFP, platelet concentrate and cryoprecipitate transfusion expressed as any vs. none and total volume of each blood component.
- Non-blood component pro-haemostatic treatments (extra protamine, rFVIIa, any antifibrinolytic drug, fibrin sealant or fibrinogen concentrate) expressed as total dose per Kg body weight of each agent.
- Time from cessation of CPB to chest closure.
- Post-operative complications or death.

### Laboratory testing

Each blood sample will undergo parallel testing by:

- Thromboelastometry (ROTEM, Tem Innovations GmbH). Four different tests will be performed on this platform (INTEM, EXTEM, FIBTEM and HEPTEM tests using manufacturer's standard reagents). Each test will yield eight parameters; i) clot time (CT), ii) clot formation time (CFT), iii) alpha angle, iv) maximum clot firmness (MCF), v-vii) amplitude at 10, 20 and 30 min after CT and viii) lysis index at 30 min after CT.
- Reference laboratory tests. These tests will provide 10 measurements on each sample;
- Prothrombin time and activated partial thromboplastin time
- Fibrinogen activity (Clauss assay)
- Anti-Xa assay (heparin activity)
- Endogenous thrombin potential ((ETP); thrombin generation assay in plasma)
- Platelet count
- Platelet function testing by whole blood electrical impedance aggregometry (Multiplate, Roche Diagnostics) with the Adenosine diphosphate (ADP-test), Thrombin Receptor Activating Peptide (TRAP-test) and Collagen (COLL-test) standard manufacturer's reagents. The platelet function test results will be expressed as area under curve, lag time and amplitude.

### Sample size calculation

The study size is 200 participants with blood samples taken. In estimating the power of the study and precision of the estimates, we have assumed that the rate of the primary outcome is 50 % in this population. Additionally, we have considered two alternative estimates of prevalence of blood clotting abnormalities (30 % and 5 %) in the study population:

**Objective 1** (describing the prevalence of different blood clotting abnormalities in the population): a sample size of 200 participants will allow a prevalence of 30 % to be estimated with a 95 % confidence interval of +/− 7 % (23.7 %, 36.9 %) and a prevalence of 5 % to be estimated with a 95 % confidence interval of +/− 4 % (2.4 %, 9.0 %).

**Objective 2** (estimating the extent to which TEM and reference laboratory test results predict clinical concern about bleeding): the power to estimate a relative risk of a given magnitude is dependent on the prevalence of the blood clotting abnormalities and increases with increasing prevalence. The study will have a minimum of 77 % power to estimate a relative risk of 1.5, given a rate of blood clotting abnormalities of >30 % in the population and >80 % power to estimate a relative risk of 2, given a rate of blood clotting abnormalities of between 4 % and 5 %.

**Objective 3** (estimating diagnostic accuracy of TEM test results vs. reference test results): a sample size of 200 participants will allow the sensitivity and specificity of each TEM test result to identify defined blood clotting abnormalities with a prevalence of 30 % to be estimated with a 95 % confidence interval of +/− 10 % (79.5 %, 96.2 %) for a sensitivity of 90 % increasing to +/−13 % (46.5 %, 72.4 %) for a sensitivity of 60 %. The specificity will be estimated with a 95 % confidence interval of +/−6 % (83.8 %, 94.4 %) for a specificity of 90 % increasing to +/−9 % (51.4 %, 68.2 %) for a specificity of 60 %. For a prevalence of 5 %, the 95 % confidence intervals are +/− 35 % (55.5 %, 100 %) for a sensitivity of 90 % and +/−35 % (26.4 %, 87.8 %) for a sensitivity of 60 %. The corresponding figures for specificity are +/− 5 % (84.8 %, 93.9 %) for 90 % specificity and +/− 7 % (52.7 %, 67.0 %) for 60 % specificity.

### Study methods

Participants will undergo standard pre-operative, anaesthetic, surgical and post-operative care according to existing institutional protocols. Decisions about the prevention or treatment of excessive bleeding during or after surgery will be guided by clinician judgement and routine laboratory investigations (such as measurement of heparin level) in accordance with our routine institutional practice. These decisions will not be influenced by study participation although clinicians will be asked to record the indication for each pro-haemostatic treatment during intervals A, B and C, as described above. Blood samples will be taken from existing vascular lines that are inserted as part of standard clinical care without the need for additional venepunctures.

In order to enable laboratory quality control and assay validation purposes, blood samples not used in the initial analyses will be stored at −80 °C for the duration of the study. Specimens that are left over upon completion of the study have the potential of being used for future research relevant to cardiac surgery. Therefore, participants or their parent/guardian are asked to give their permission for samples to be retained after the end of the study for use in other ethically approved research. The samples of participants who do not consent to this option will be destroyed at the end of this study in accordance with the Human Tissue Authority’s Code of Practice. The responsibility for custodianship of samples retained beyond the end of this study will lie with the Chief investigator.

Data collection will include the following elements:

- A log of all children having cardiac surgery
- A log of patients assessed against the eligibility criteria and, if ineligible, reasons for ineligibility.
- A log of potential participants or parents/guardians approached for the study (including the date when they are given the Patient Information Leaflet).
- Consent and baseline information, including demographic characteristics, clinical history, planned procedure.
- Data characterising the participant’s hospital stay
- Operative details
- Clinical observations and routinely collected laboratory test results
- Post-operative care
- Post-operative complications
- Blood clotting abnormality test results collected as part of the study will be obtained from the ROTEM device hard-drive, Multiplate device hard-drive, ETP device hard-drive, and for other tests, from the Bristol University Hospitals NHS Foundation Trust (UHBristol) electronic laboratory database.
- Data describing red blood cell, platelet and fresh frozen plasma transfusions will be obtained from the UHBristol blood bank electronic laboratory database.
- Data describing all other pro-haemostatic treatments.

A large amount of data will be available from routine sources such as medical records and intra/post-operative care charts. Study data will be recorded on a study CRF before transfer to a custom made electronic database held on a secure server. Study-specific CRFs will be used as required.

### Statistical analyses

In order to address objective 1 (describing the prevalence of the different types of blood clotting abnormality before and after cardiac surgery) the following analyses will be undertaken:

- The distributions of the measurements from each reference laboratory test will be characterised separately for the two sample time-points.
- Principal component, latent class or similar analyses will be selected depending on the nature of the distributions and will be used to define and describe blood clotting abnormalities on the basis of the reference laboratory test results.
- The proportions of participants with different blood clotting abnormalities based on the classification determined in b) and with no blood clotting abnormality will be described with 95 % confidence intervals.

Logistic regression models will be fitted to estimate extent to which the individual results of the TEM and reference laboratory test results at each time point predict clinical concern about bleeding (objective 2a). Receiver operator characteristic (ROC) analyses will be performed to identify optimal cut-off criteria for predicting clinical concern about bleeding using separate analyses for each category of blood clotting abnormalities. The most prevalent clusters of abnormal reference laboratory test results (identified in objective 1) will be represented as semantic diagnostic terms representing different possible blood clotting abnormalities (e.g. increased APTT and increased anti-Xa heparin activity, but normal other reference laboratory tests indicates "residual heparin") and associations between these terms and clinical concern about bleeding will be investigated using logistic regression models (objective 2b).

We will estimate the diagnostic accuracy of TEM test results for the different types of blood clotting abnormality defined by the results of the reference laboratory tests (objective 3) by calculating the sensitivity and specificity, likelihood ratios and positive and negative predictive values for different estimates of the prevalence of blood clotting abnormality.

The agreement between the recommended treatments resulting from the reference tests and the TEM test results will be assessed using Kappa statistics (objective 4). The proportion of participants correctly identified by diagnostic models using either TEM or reference test results will be subject to ‘optimism’ bias. Ideally, this would be addressed by validating models on ‘new’ data that is not used for developing the models (‘train-and-test’ validation). Since the sample size available for this study is not large enough to permit this, we will consider alternative methods such as cross-validation and boot-strapping.

Analyses relating to objectives 1, 2 and 3 above may additionally be stratified by age group. The primary analysis will take place when all data have been collected, samples analysed and the database is locked. No interim analysis is planned.

## Discussion

Since the DECISION study was conceived, the National Institute of Clinical Excellence (NICE) have issued guidance about the implementation of TEM and other rapid viscoelastometric tests in clinical practice, including cardiac surgery [10]. The guidance states that TEM are recommended to help monitor blood clotting during and after heart surgery by healthcare professionals who have had appropriate training. The Committee recommended further research comparing the clinical effectiveness of the three alternative commercial viscoelastometric devices (ROTEM, TEG and Sonoclot systems) in cardiac surgery to determine which test parameters are most useful for informing treatment of excessive bleeding caused by blood clotting abnormalities. This guidance is based on a systematic review that excluded paediatric studies, [10, 7] highlighting that there is still an unmet need to evaluate the clinical utility of TEM testing in children.

### Trial status

The trial opened to recruitment in May 2013 and it is planned that the recruitment will be completed by May 2015. Recruitment is currently ahead of target.

## Abbreviations

ADP-test: Multiplate platelet function test utilising the Adenosine diphosphate reagent
CFT: clot formation time
COL-test: Multiplate platelet function test utilising the collagen reagent
CPB: Cardiopulmonary bypass
CRF: Case report form
CT: clot time
ETP: Endogenous Thrombin Potential
EXTEM: ROTEM test utilising tissue factor activator
FFP: Fresh frozen plasma
FIBTEM: ROTEM test utilising tissue factor activator, modified to reflect fibrinogen activity
HEPTEM: ROTEM test utilising contact activator, modified to reflect heparin activity
INTEM: ROTEM test utilising contact activator
MCF: maximum clot firmness
rFVIIa: Recombinant factor 7
rFVIIa: Recombinant factor 7
ADP-test: Multiplate platelet function test utilising the Adenosine diphosphate reagent
COL-test: Multiplate platelet function test utilising the collagen reagent
CPB: Cardiopulmonary bypass
CRF: Case report form
ETP: Endogenous Thrombin Potential
EXTEM: ROTEM test utilising tissue factor activator
FFP: Fresh frozen plasma
FIBTEM: ROTEM test utilising tissue factor activator, modified to reflect fibrinogen activity
HEPTEM: ROTEM test utilising contact activator, modified to reflect heparin activity
INTEM: ROTEM test utilising contact activator
MCF: maximum clot firmness
NICE: National Institute of Clinical Excellence
rFVIIa: Recombinant factor 7
ROC: Receiver operating characteristic
ROTEM: Rotational thromboelastometry
TEM: Thromboelastometry
TRAP-test: Multiplate platelet function test utilising thrombin receptor activating peptide
UHBristol: University Hospitals Bristol NHS Foundation Trust

## References

[1] Szekely A, Cserep Z, Sapi E, Breuer T, Nagy CA, Vargha P (2009). Risks and predictors of blood transfusion in pediatric patients undergoing open heart operations. Ann Thorac Surg.

[2] New HV, Kelleher A, Arnold P, Ng C, Hennem S, Hall T, et al. A mulitcentre prospective observational survey of the perioperative use of blood components in paediatric cardiac surgery. Br J Haematol. 2011;153(supplement s1):11.

[3] Miller BE, Mochizuki T, Levy JH, Bailey JM, Tosone SR, Tam VK (1997). Predicting and treating coagulopathies after cardiopulmonary bypass in children. Anaesth Analg.

[4] Williams GD, Bratton SL, Riley EC, Ramamoorthy C (1999). Coagulation tests during cardiopulmonary bypass correlate with blood loss in children undergoing cardiac surgery. J Cardiothorac Vasc Anesth.

[5] Moganasundram S, Hunt BJ, Sykes K, Holton F, Parmar K, Durward A (2010). The relationship among thromboelastography, hemostatic variables, and bleeding after cardiopulmonary bypass surgery in children. Anaesth Analg.

[6] Shore-Lesserson L, Manspeizer HE, DePerio M, Francis S, Vela-Cantos F, Ergin MA (1999). Thromboelastography-guided transfusion algorithm reduces transfusions in complex cardiac surgery. Anaesth Analg.

[7] Ak K, Isbir CS, Tetik S, Atalan N, Tekeli A, Aljodi M (2009). Thromboelastography-based transfusion algorithm reduces blood product use after elective CABG: a prospective randomized study. J Card Surg.

[8] Despotis GJ, Santoro SA, Spitznagel E, Kater KM, Cox JL, Barnes P (1994). Prospective evaluation and clinical utility of on-site monitoring of coagulation in patients undergoing cardiac operation. J Thorac Cardiovasc Surg.

[9] Weber CF, Gorlinger K, Meininger D, Herrmann E, Bingold T, Moritz A (2012). Point-of-care testing: a prospective, randomized clinical trial of efficacy in coagulopathic cardiac surgery patients. Anesthesiology.

[10] http://www.nice.org.uk/guidance/dg13. Detecting, managing and monitoring haemostasis: viscoelastometric point of care testing (ROTEM, TEG and Sonoclot systems) 2014.

